# Supplementary material for: Unbiased proteomics, histochemistry, and mitochondrial DNA copy number reveal better mitochondrial health in muscle of high-functioning octogenarians
Source: eLife. 2022 Apr 11;11:e74335. doi: 10.7554/eLife.74335 (PMC9090325; doi:10.7554/eLife.74335)
Supplement: Figure 3—figure supplement 2—source data 2. [file elife-74335-fig3-figsupp2-data2.pdf]

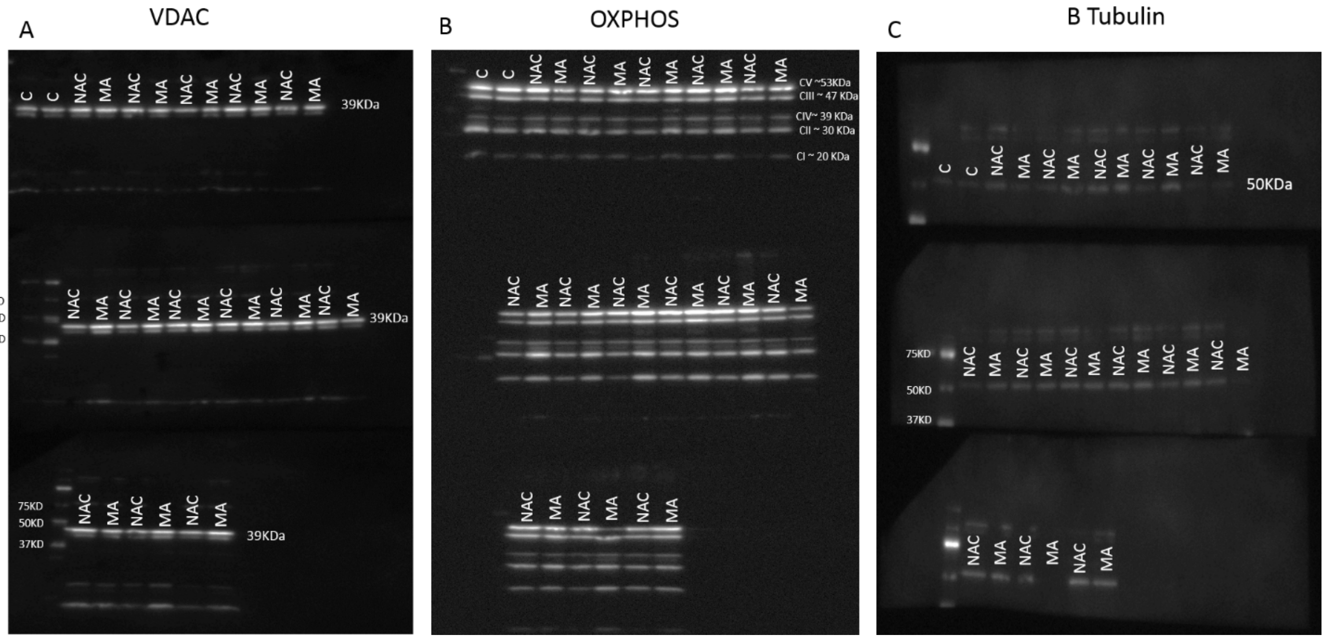

Figure 3 –figure supplement 2-source data 1. Mitochondrial protein quantification. Mitochondrial protein quantification was performed by immunoblotting of the mitochondrial proteins VDAC (**A**) and the components of the OXPHOS system (**B**). The samples were loaded alternatively i.e. one MA and one NA. Gels held 12 samples plus a pre stained protein standard (161-0375 BioRad). Quantification was performed on images taken with all three gels present. The gels were probed for tubulin (**C**), stripped, then probed for VDAC, stripped again and then probed for the OXPHOS subunits. C = samples of protein from subjects not included in this study.
