# Supplementary material for: Unbiased proteomics, histochemistry, and mitochondrial DNA copy number reveal better mitochondrial health in muscle of high-functioning octogenarians
Source: eLife. 2022 Apr 11;11:e74335. doi: 10.7554/eLife.74335 (PMC9090325; doi:10.7554/eLife.74335)
Supplement: Figure 5—source data 1. — The participants are classified into five different age groups. Gender: the number of donors is represented in numeric, M is male, F is female. Age in years as mean and standard deviation (SD±) for each age group. Race: number of donors is shown in left and race is shown in italics, C is Caucasian, AA is African American, and A is Asian. Body mass index (BMI) expressed as mean and SD (±) for each group. p-Value is calculated by one-way ANOVA with Kruskal–Wallis test. *p-Value calculated from linear regression model, gender adjusted. ± knee extension isokinetic strength (KEIS) (300/s; Nm). †Physical activity is calculated from the sum of weight circuit, vigorous exercise, brisk walking and casual walking, and summed as high-intensity physical activity per week. This is further categorized into 0 (not active), 1 (moderately active), 2 (active), and 3 (highly active) and expressed as mean of categorical variables (0, 1, 2, 3) ± SD. [file elife-74335-fig5-data1.zip › Figure_5XXXfigure_supplement_1 (copy).pdf]

Figure 5–figure supplement 1

**Age Group**	**20-34**	**35-49**	**50-64**	**65-79**	**80+**	***P*-value**	**R^2**
	(*n*=13)	(*n*=11)	(*n*=12)	(*n*=12)	(*n*=10)	--	--
**Gender**	*M*8, *F*5	*M*7, *F*4	*M*7, *F*5	*M*8, *F*4	*M6*, *F4*	--	--
**Age (yr)**	27.2 ± 3.3	41.3 ± 4.5	57.1 ± 4.7	70.3 ± 2.3	82.4 ± 2.4	--	--
**Race**	9*C*, 2*AA*, 2*A*	5*C*, 6*AA*	8*C*, 4*AA*	10*C*,1*AA*,1*A*	9*C*,1*AA*	0.0958	--
***BMI, kg/m^2**	25.9 ± 2.8	26.4 ± 2.6	26.6 ± 3.2	26.4 ± 2.4	25.2 ± 3.9	0.3458	0.007
**Height (cm)**	172 ± 11	177 ± 10	169 ± 4	172 ± 11	172 ± 6	0.3985	--
***Weight (kg)**	76 ± 10	81 ± 9	77 ± 12	75 ± 13	73 ± 16	**1.74E-05**	0.34
**Education (yr)**	16 ± 3	14 ±3	14 ± 2	16 ± 2	17 ± 2	0.3305	--
***Waist Circumference(cm)**	82 ± 7	87 ± 7	90 ± 11	92 ± 11	92 ± 13	**6.32E-06**	0.39
***KEIS (left) ±**	192 ± 31	208 ±55	200 ± 71	165 ± 62	130 ± 42	**4.29E-07**	0.40
***KEIS (right) ±**	194 ± 38	220 ± 65	194 ±78	169 ± 53	147 ± 57	**2.41E-07**	0.41
**†Physical Activity**	1.8 ± 1.4	1.8 ± 1.3	2 ± 1.1	2.3 ± 1	1.5 ± 1.1	0.5145	--

Figure 5–figure supplement 1. Baseline characteristics of the GESTALT skeletal muscle participants. The participants are classified into 5 different age groups. Gender: The number of donors is represented in numeric, *M* is Male, *F* is Female. Age in years as mean and standard deviation (SD ±) for each age group. Race: number of donors is shown in left and race is shown in italics, *C* is Caucasian, *AA* is African American, and *A* is Asian. Body Mass Index (BMI) expressed as mean and SD (±) for each group. *P*-value is calculated by 1-way ANOVA with Kruskal-Wallis test.

**P*-value calculated from linear regression model, gender adjusted.

± Knee Extension Isokinetic Strength (KEIS) (30^0/sec; Nm).

†Physical activity is calculated from the sum of weight circuit, vigorous exercise, brisk walking and casual walking and summed as high intensity physical activity per week. This is further categorized into 0 (not active),1 (moderately active), 2 (active), and 3 (highly active) and expressed as mean of categorical variables (0,1,2,3) ± SD.
